# Retraction Note: Histone methyltransferase SUV39H2 regulates LSD1-dependent CDH1 expression and promotes epithelial mesenchymal transition of osteosarcoma

**DOI:** 10.1186/s12935-023-03016-3

**Published:** 2023-08-18

**Authors:** Yingying Miao, Guifeng Liu, Lin Liu

**Affiliations:** 1 Department of Anesthesiology, Union Hospital of Jilin University, Changchun, 130033 People’s Republic of China; 2 Department of Radiology, Union Hospital of Jilin University, No. 126, Xiantai Street, Changchun, 130033 Jilin People’s Republic of China

**Retraction Note: Cancer Cell International (2021) 21:2**

10.1186/s12935-020-01636-7

The Editors-in-Chief have retracted this article at the request of the authors. The first corresponding author has stated that the authors found that the experiments and results could not be repeated. Additionally, there appear to be discrepancies between the documents for the animal ethics approval and what is reported in the article. The Editors-in-Chief therefore no longer have confidence in the results and conclusions reported in this article.

The authors agree with this retraction.

